# The cell origins of foam cell and lipid metabolism regulated by mechanical stress in atherosclerosis

**DOI:** 10.3389/fphys.2023.1179828

**Published:** 2023-04-13

**Authors:** Zhi Ouyang, Jian Zhong, Junyi Shen, Ye Zeng

**Affiliations:** Institute of Biomedical Engineering, West China School of Basic Medical Sciences & Forensic Medicine, Sichuan University, Chengdu, China

**Keywords:** atherosclerosis, foam cell, lipid metabolism, mechanical force, endothelial cell

## Abstract

Atherosclerosis is an inflammatory disease initiated by endothelial activation, in which lipoprotein, cholesterol, extracellular matrix, and various types of immune and non-immune cells are accumulated and formed into plaques on the arterial wall suffering from disturbed flow, characterized by low and oscillating shear stress. Foam cells are a major cellular component in atherosclerotic plaques, which play an indispensable role in the occurrence, development and rupture of atherosclerotic plaques. It was previously believed that foam cells were derived from macrophages or smooth muscle cells, but recent studies have suggested that there are other sources of foam cells. Many studies have found that the distribution of atherosclerotic plaques is not random but distributed at the bend and bifurcation of the arterial tree. The development and rupture of atherosclerotic plaque are affected by mechanical stress. In this review, we reviewed the advances in foam cell formation in atherosclerosis and the regulation of atherosclerotic plaque and lipid metabolism by mechanical forces. These findings provide new clues for investigating the mechanisms of atherosclerotic plaque formation and progression.

## Introduction

Atherosclerosis (AS) is a chronic disease that begins early in life and then persists throughout life (Zeng, 2017; Yao et al., 2020; Zeng and Fu, 2021). Vascular remodeling involves a combination of vascular endothelial dysfunction, extensive intimal lipid deposition, enhanced innate and adaptive immune responses, vascular smooth muscle cell (VSMC) proliferation and extracellular matrix (ECM) remodeling, culminating in the atherosclerotic plaques formation and cardiovascular disease promotion (Badimon and Vilahur, 2014). The formation and accumulation of foam cells is a critical process of intimal lipid deposition during atherosclerosis (Maguire et al., 2019), which plays an important role in all stages of atherosclerotic lesion development, from the initial lesion to the advanced plaque. Considering the cholesterol level is closely related to the risk of cardiovascular disease, many lipid-lowering agents have been in clinical use, such as statins, bile acid sequestrants, ezetimibe, fibrates, omega-3 fatty acids, niacin, proprotein convertase subtilisin/kexin type 9 (PCSK9) inhibitors and dietary supplements (Zivkovic et al., 2023). However, treatment failure and recurrent cardiovascular events occurred frequently (Kim et al., 2022). Novel strategies to control lipid deposition without affecting inflammation and immunomodulation are desired.

Recent single-cell RNA-seq and single-cell ATAC-seq of human carotid plaques identified 14 different cell populations, including endothelial cells (ECs), VSMCs, mast cells, B cells, myeloid cells, and T cells (Depuydt et al., 2020). It is generally believed to be the result of uncontrolled uptake of oxidative low-density lipoprotein (ox-LDL) by macrophages. Excessive cholesterol esterification and/or impaired cholesterol release in macrophages leads to the storage as cytoplasmic lipid droplets, which initiates the formation of foam cells (Yu et al., 2013). There is a crosstalk between macrophage and VSMCs. VSMCs are key participants in early and advanced atherosclerosis. VSMCs invade the early atherosclerotic lesions from the media to enlarge the lesion, and they are also able to form a protective ECM-rich fibrous cap that covers the necrotic core. However, VSMCs can differentiate into many different phenotypes *in vivo*, including macrophage-like, foam-like, osteochondrogenic-like, myofibroblast-like, and mesenchymal stem cell-like phenotypes (Grootaert and Bennett, 2021). For example, VSMCs can transdifferentiate into macrophage-like cells that involved in the necrotic core formation (Xue et al., 2022; Zhang et al., 2022). Recently foam cells in plaques were shown to express not only CD36 and α-SMA, but also CD31 and S100 (Ru et al., 2022). It implies that ECs are another origin of foam cells. This view could be supported by the uptake of lipoproteins by ECs (Baumer et al., 2017). However, their contribution to foam cells is rarely investigated (Gui et al., 2022).

**FIGURE 1:**
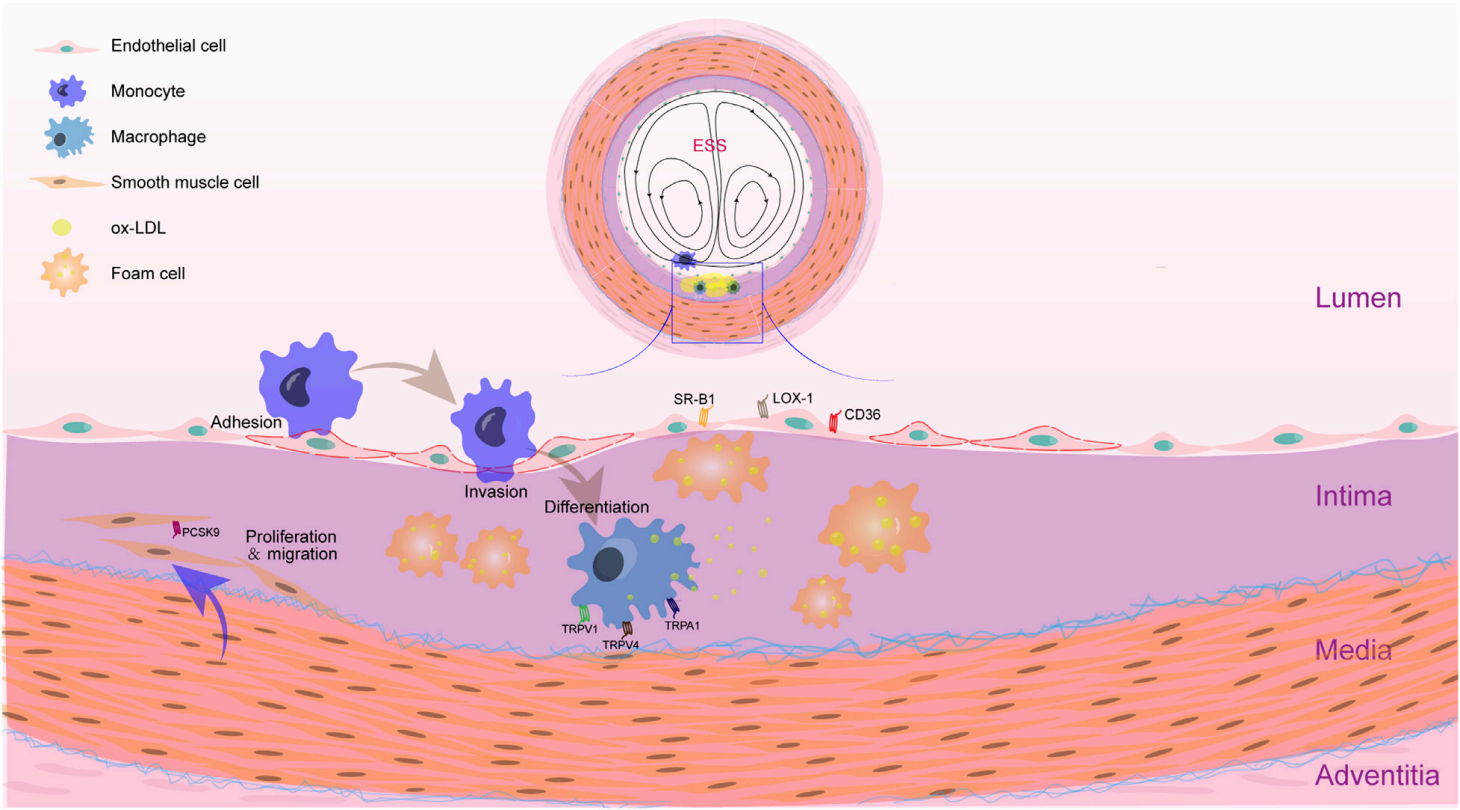
Feature of AS plaque in the location with disturbed shear stress. The accumulation of foam cells is an essential process in the formation of atherosclerotic plaque. Within the intima of blood vessels with disturbed shear stress, ox-LDL accumulated. Monocytes adhere to endothelial cells and invade into the intima. Then, monocytes differentiate into macrophages and phagocytose ox-LDL. When the phagocytosis of ox-LDL by macrophages increases and the efflux is blocked, foam cells will be formed. VSMCs that are proliferated and migrated into the intima can also uptake the ox-LDL. Recent studies have shown that many receptors related to lipid metabolism in macrophages, VSMCs and ECs are mechanosensors, such as TRPV1, TRPV4 and TRPA1 in macrophages, PCSK9 in VSMCs, and SR-B1, LOX-1 and CD36 in ECs. These elements may influence the lipid metabolism in response to altered mechanical forces.

Many studies have shown that mechanical forces play an important role in the development of atherosclerotic plaques. Mechanical forces are necessary for the embryogenesis and maintenance of physiological functions of various vascular cells, including aortic endothelial cells, resident macrophages, and VSMCs (Mukherjee et al., 2022). Atherosclerotic plaques are not randomly distributed on the arterial wall, but are preferentially located at the bends and bifurcations of the arterial tree suffering from disturbed flow, characterized by low and oscillating shear stress (He et al., 2022). Shear stress is altered with the progress of atherosclerotic plaque and narrowing of the vascular lumen. As a major component of atherosclerotic plaques, foam cells contribute to cholesterol deposition and plaques growth (Figure 1). The cell origin of foam cells and lipid metabolism pathways, as well as the regulatory role of shear stress are focused in the present review and some issues are proposed. The role of ECs in the formation of foam cells and the effect of mechanical forces on cell foaming are highlighted.

## Origin of foam cells

Atherosclerosis is a chronic immunoinflammatory and fibroproliferative disease of medium and large arteries caused by lipids (Falk, 2006). Foam cells play an important role in the development of atherosclerosis. Current studies suggest that there are three sources of foam cells, including macrophages, VSMCs, and ECs (Chistiakov et al., 2017).

### Macrophage-derived foam cell

There are three processes of lipid metabolism in macrophages: cholesterol uptake, esterification, and efflux. However, during atherosclerosis, dysregulation of lipid metabolic pathways leads to the accumulation of foam cells in the intima of arteries (Maguire et al., 2019). As these foam cells accumulate, they play a role in promoting atherosclerosis. When monocytes enter the endothelium, they differentiate into macrophages. Macrophages can be classified into many different types based on their phenotype versus their functional role in the plaque. One of the classical inflammatory macrophage phenotypes is named M1, which can be induced by a combination of interferon-γ (INF-γ) and toll-like receptor 4 (TLR4) ligand lipopolysaccharide in macrophages cultured *in vitro*. *In vitro*, there are several subsets of macrophages that are activated alternately, called M2 macrophages, which can be induced by culturing macrophages with IL-4 and IL-13 (Tabas and Bornfeldt, 2016). In addition, there are several macrophages that play a role in atherosclerotic lesions. These include the M(Hb) and Mhem populations (Boyle et al., 2009), which are induced *in vitro* by hemoglobin-haptoglobin complexes and heme, and are resistant to lipid loading. *In vivo*, Mox macrophages induced by oxidized phospholipids are characterized by high expression of heme oxygenase, while M4 macrophages were induced by chemokine CXCL4 (Li et al., 2022). Nowadays, neither M1 nor M2 type macrophages have been identified as precursors of foam cells (Stöger et al., 2012). However, it has been shown that anti-inflammatory M2 macrophages are more likely to form foam cells than M1 macrophages (van Tits et al., 2011).

Modified LDL, aggregated lipoproteins, and other substances can be taken up by various types of scavenger receptors (SRs) in macrophages, such as Class A SR (SR-A), CD36, lectin-like ox-LDL receptor-1 (LOX-1), and low-density lipoprotein receptor-related protein-1 (LRP-1) (Chistiakov et al., 2016). Among them, SR-A-like receptors, including SR-AI and SR-AII, mainly take up acetylated LDL, but have a lower affinity for ox-LDL (Moore and Freeman, 2006). Suzuki et al. (1997) showed that deletion of SR-A receptors in macrophages reduces the uptake of modified LDL.

Another scavenger receptor involved in macrophage-derived foam cell formation is CD36. Unlike SR-AI and SR-AII, CD36 does not have a high affinity for acetylated LDL or extensively oxidized LDL, and CD36 is distributed in many cells, including monocytes, macrophages, adipocytes, ECs, and other cells (Moore and Freeman, 2006). Abnormal upregulation of CD36 can promote inflammation, foam cell formation, and EC apoptosis, but CD36 deficiency can also lead to dyslipidemia and metabolic disorders (Zhao et al., 2018). Upregulation of miRNA by macrophage NFATc3 reduces the levels of SR-A and CD36, thus preventing the formation of foam cells (Liu et al., 2021).

The third scavenger receptor is LOX-1, which is the major ox-LDL receptor in ECs and is also expressed in macrophages and VSMCs (Pirillo et al., 2013). The expression of LOX-1 in macrophages can inhibit the migration of macrophages and increase the foaming of macrophages (Kattoor et al., 2019). *In vitro*, LOX-1 was upregulated in macrophages in response to inflammatory stimuli (Kume et al., 2000). In addition, the plasma membrane scavenger receptor LRP1 also plays an important role in the atherosclerotic process. Cholesterol has been shown to regulate the extracellular structural domain of LRP1, thereby regulating LRP1 levels and plasma membrane function (Selvais et al., 2011). Apolipoprotein E expression is increased in LRP1-deficient macrophages (Yancey et al., 2010), suggesting that the LRP1/apoE axis can regulate apoptosis and thus prevent necrotic core formation.

After being phagocytosed, the modified lipoproteins enter the intracellular lysosomes and are then hydrolyzed into free cholesterol and fatty acids. In which the conversion of free cholesterol to cholesterol esters is necessary for the formation of macrophage-derived foam cells. Cholesterol acyltransferase (ACTA) plays an important role in cholesterol esterification. ACAT-1 catalyzes the formation of cholesterol esters from cholesterol and long-chain fatty acyl-CoA. The accumulation of cholesterol esters produced by ACAT-1 in macrophages plays an important role in the formation of foam cells under pathological conditions (Chang et al., 2006). It has been shown that inhibition of ACAT-1 gene expression using the dipeptidyl peptidase-4 (DPP-4) inhibitor teneligliptin can inhibit the formation of macrophage-derived foam cells (Terasaki et al., 2020). Fujiwara et al. (2007) gave ApoE−/− mice hesperidin to inhibit the activity of ACAT-1 and ACAT-2 proteins and found a decrease in serum levels of cholesterol, triglyceride and LDL-cholesterol and atherosclerotic lesion area in those mice. In addition, neutral cholesteryl ester hydrolase (NCEH) is essential for cholesterol efflux. In contrast to ACAT-1, NCEH produces free cholesterol and fatty acids (Sekiya et al., 2011). Upon silencing of NCEH, cholesteryl esters accumulate in macrophages; conversely, when NCEH is overexpressed, cholesteryl ester formation in macrophages is inhibited (Okazaki et al., 2008).

The final step in macrophage cholesterol metabolism is the efflux of cholesterol. Active efflux of cholesterol is mainly mediated by ATP-binding cassette (ABC) transporters ABCA1 and ABCG1. ABCA1 is necessary for the transport of cholesterol and phospholipids to apolipoprotein -I and the biosynthesis of HDL; while ABCG1 mainly promotes the efflux of cholesterol to HDL (Wang and Westerterp, 2020). When ABCA1 expression is increased in macrophages, cholesterol efflux is increased, and foam cell formation is reduced (Price et al., 2019). Conversely, when ABCA1 and scavenger receptor class B type 1 (SR-B1) are knocked out, mice developed severe hypercholesterolemia along with enhanced foam cell accumulation (Zhao et al., 2011). However, in LDLR−/− mice fed a high-fat diet, deletion of ABCG1 reduces atherosclerotic lesions because ABCG1 promoted cholesterol accumulation and alters the morphology of T cells (Cheng et al., 2016). Finally, SR-B1 is a multiligand membrane receptor protein that promotes cholesterol influx from macrophages into the liver (Shen et al., 2018). After constructing SR-B1-deficient mice in LDLR−/− mice, aortic atherosclerosis formation was found to be accelerated in these mice (Galle-Treger et al., 2020). This is due to the increased apoptosis of macrophages as a result of SR-B1 deficiency (Tao et al., 2021).

### VSMC-derived foam cell

VSMCs play an important role in all stages of atherosclerosis and are the main source of intraplaque cells and extracellular matrix (ECM). Diffuse intimal thickening (DITs) is the most initial stage of atherosclerosis, they are formed from birth. The first stage of atherosclerosis is PITs (pathological intimal thickening), where extracellular lipid pools are formed deep in the intima under VSMCs and ECM (Virmani et al., 2000). During the transition from DITs to PITs, LDL in the circulating blood remains in the intima due to the interaction of LDL apolipoprotein with proteoglycans produced by VSMCs, which taken up ox-LDL and formed foam cells (Basatemur et al., 2019). Like macrophages, VSMCs employ a similar strategy for treating modified lipoproteins in their environment. Furthermore, the phenotypic transformation of VSMCs plays an important role, with the co-expression of αSMA and CD68 detected in atherosclerotic plaques (Andreeva et al., 1997). Single-cell RNA-seq of VSMCs showed that VSMCs were transferred into “SEM” cells (stem cells, ECs, and monocyte) during atherosclerosis. The SEM cells were multipotent and could differentiate into macrophage-like and fibrochondrocyte-like cells, as well as return toward the VSMC phenotype (Pan et al., 2020). Compared with the mesothelial VSMCs, VSMCs in DITs have more synthetic organelles (Mosse et al., 1985). This phenomenon indicates a shift towards synthetic phenotypes in VSMCs in DITs. Allahverdian *et al* showed that the expression of ABCA1 was lower in synthetic VSMCs in the intima than those in the tunica media (Allahverdian et al., 2014).

There are many VSMCs-derived cells expressing macrophage markers in the intima of atherosclerotic ApoE−/− mice (Feil et al., 2014), suggesting that VSMCs switch to lipid-rich macrophage-like cells during atherosclerosis.

Krüppel-like factor 4 (KLF-4) plays a key role in the phenotypic transition of VSMCs. Shankman et al. (2015) showed that in ApoE−/− mice in which KLF-4 was VSMCs-specific conditional knocked out, the number of macrophage-like cells and the size of atherosclerotic lesions were decreased. Furthermore, ox-LDL reduced the expression of VSMC markers by activating monocytes and upregulated the expression of KLF-4 and macrophage markers (Burger et al., 2021). ECM is also important for the maintenance of the contractile phenotype of VSMCs, and Nidogen-2 in the ECM can maintain the contractile phenotype of VSMCs via Jagged1-Notch3 (Mao et al., 2021). In addition, lipid metabolism of VSMCs has also been studied. In ApoE−/− mice, overexpression of Wnt5a increased cholesterol accumulation in VSMCs by inhibiting the expression of ABCA1 (Zhang et al., 2020). Other studies have shown that VSMCs respond to ox-LDL stimulation via TLR4, which regulates ABCG1 expression and subsequently induces lipid accumulation in VSMCs via the PPARγ/LXRa signaling pathway (Cao et al., 2017).

### EC-derived foam cell

Studies on the EC-derived foam cells are scarce, but the expression of SRs on ECs is changed in lipid metabolism. Studies have shown that high-fat fed ECs-CD36-knocked out (KO) mice have reduced insulin sensitivity and uptake of fatty acids by heart and skeletal muscle (Son et al., 2018). LOX-1 is predominantly expressed in ECs. The endothelial-specific overexpressed LOX-1 leads to the increased phosphorylation of P38 and the increased activity of NF-κB, which is conducive to atherosclerosis (Akhmedov et al., 2014). Furthermore, in C57BL/6 mice fed with high-fat diet, overexpression of SR-B1 reduced atherosclerosis (Vaisman et al., 2015). However, endothelial-specific knockout of SR-B1 reduced atherosclerotic plaques in ApoE−/− mice and in PSCK9-induced LDLR−/− mice (Huang et al., 2019). *In vitro*, studies have shown that SR-B1 and ABCG1 of ECs are important for HDL transport (Rohrer et al., 2009).

After treating ECs with human hyperlipidemia serum, Ivan *et al* found that ECs began to load lipid droplets and transform into foam cells (Ivan and Antohe, 2010). Although studies have shown that ECs express several lipid receptors, little research has been done on the transformation of ECs into foam cells. More experiments *in vitro* and *in vivo* are needed to investigate the transition mechanism, which is very important for finding targets for the treatment of atherosclerosis.

**FIGURE 2:**
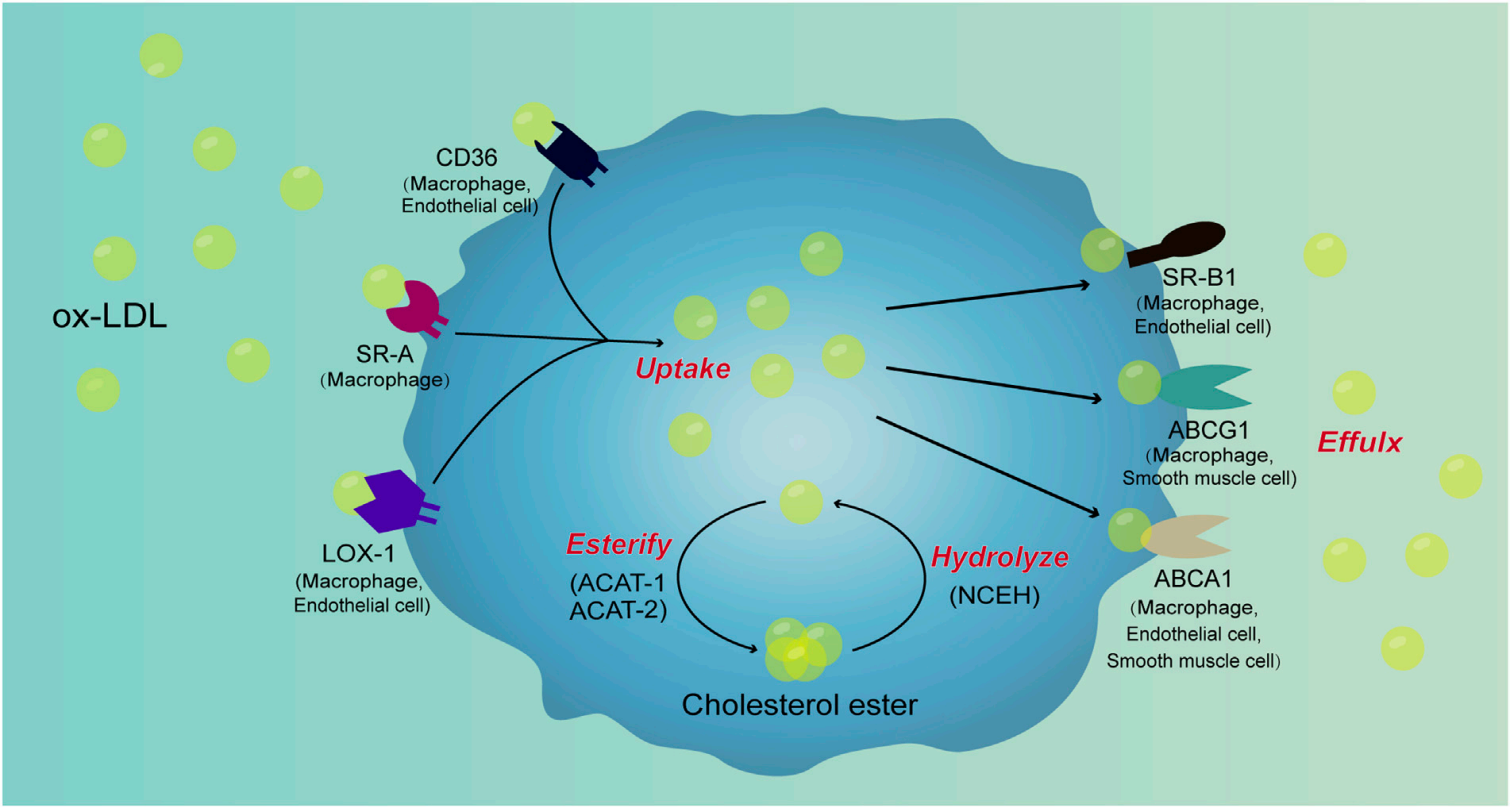
Signal pathways involved in cell foaming. Ox-LDL is taken up by macrophages through scavenger receptors such as CD36, SR-A, and LOX-1. After uptake of ox-LDL, a portion of ox-LDL is converted to cholesterol esters by ACAT-1 and ACAT-2, cholesterol esters are hydrolyzed to ox-LDL by NCEH. Ox-LDL produced by the hydrolysis of cholesterol esters is excreted from the cells along with free ox-LDL in the cells through receptors such as SR-B1, ABCG1, and ABCA1. When the lipid uptake of macrophages is not controlled, the production of cholesterol esters increases and hydrolysis and efflux are blocked, cholesterol esters will accumulate in cells to form foam cells. There are scavenger receptors such as CD36, SR-A, LOX-1, SR-B1, ABCG1 and ABCA1 in macrophages. In VSMCs, ABCA1 and ABCG1 are present, while CD36, LOX-1, SR-B1 and ABCG1 are present in ECs. However, the mechanism of lipid metabolism of VSMCs and ECs and their contribution in foam cell formation require further investigation.

Therefore, the receptors involved in the uptake and efflux of lipids are presented in macrophages, VSMCs, as well as ECs (Figure 2). SRs such as CD36, SR-A, LOX-1, SR-B1, ABCG1 and ABCA1 expressed in macrophages. In VSMCs, ABCA1 and ABCG1 are present, while CD36, LOX-1, SR-B1 and ABCG1 are present in ECs. The abnormal lipid metabolism mechanism associated with foam cell formation require further investigation.

## Regulation of mechanical force on atherosclerotic plaques

Mechanical forces can play a role in the regulation of cellular physiology, thus affecting the atherosclerotic plaques progression (Hahn and Schwartz, 2009). Mechanical forces acting on the arteries are mainly fluid shear stress, circumferential stretch, and hydrostatic pressure.

Mechanical forces play an important role in the occurrence and development of atherosclerotic plaques. Low or oscillating endothelial shear stress sites near the branch point and along the inner curve are the most vulnerable (Caro et al., 1969), such as the aortic arch, carotid bifurcation, and so on. Consistently, fat streak and early plaque observed by Caro et al. (1969) was mostly distributed in the low or oscillating endothelial shear stress sites.

Partial ligation of the left carotid artery in ApoE−/− mice results in slow and disordered blood flow, increasing the plaque formation and lipid deposition in the carotid intima (Samady et al., 2011; Merino et al., 2013), with higher triglyceride levels in ligated carotid arteries compared to unligated carotid arteries (Tanaka et al., 2013). In ApoE−/− mice with impaired and partially ligated left carotid arteries, there was an increase in the content of hemolytic phosphatidylcholine and unsaturated phosphatidylcholine in the carotid artery, as well as an increase in plasma myeloperoxidase (MPO) levels and ox-LDL infiltration (Greig et al., 2015).

However, high wall shear stress (WSS) plays an important role in plaque instability. Studies by Gijsen et al. (2008) have shown that fibrous caps exposed to high WSS plaques continue to weaken over time, eventually leading to their rupture. Other studies have also shown that high WSS segments develop larger necrotic cores and calcium progression, degradation of fibrous and fibro-adipose tissue, and excessive swelling remodeling, suggesting a shift to a more fragile phenotype (Samady et al., 2011).

### Regulation of mechanical force on lipid metabolism in vascular cells

Mechanosensing is involved in fundamental cellular response processes under a variety of physiological and pathological conditions, with cells constantly adapting to changes in the external environment (Chen et al., 2017). During foam cell formation, mechanical force may play an important role. This section focuses on the regulatory role of mechanical force in lipid uptake by cells in the plaque.

### Lipid uptake by macrophages

First, the effect of sustained low shear stress leads to an increase in endothelial permeability and enhances monocyte chemotactic protein-1 (MCP-1) expression and macrophage recruitment (Ding et al., 2010), which is essential for the development of atherosclerosis.

Second, the phenotypic transformation of macrophages that is regulated by mechanical forces is important for their transformation into foam cells. Low shear stress can upregulate the pro-inflammatory mediators in atherosclerotic lesions, such as C-reactive protein, IL-6, and CXCL1, indicating that low shear stress can induce the formation of M1-type macrophages by initiating the pro-inflammatory process (Cheng et al., 2006). Low shear stress also led to the development of M1-type macrophages in high-fat fed ApoE−/− mice, whereas oscillating shear stress (OSS) caused the development of M2-type macrophages (Seneviratne et al., 2015). Studies on human atherosclerotic plaques have shown that M1-type macrophages are more likely to appear in the shoulder region of the plaques, whereas M2-type macrophages are more likely to appear in the adventitia (Stöger et al., 2012), which may be respectively controlled by shear stress and local circumferential stress of the plaques.

In addition, high flow shear stress (25 dyn/cm^2^) can induce increased phagocytosis and pro-inflammatory responses in macrophages (Rosenson-Schloss et al., 1999). The transient receptor potential (TRP) superfamily is a mechanical-sensor closely associated with atherosclerosis. The activation of TRPV1 allows macrophages to resist ox-LDL-induced lipid accumulation and reduces foam cell formation (Zhao et al., 2013). In addition, the deficiency of TRPV4 also inhibited the ox-LDL-induced formation of macrophage-derived foam cells (Goswami et al., 2017). Finally, TRPA1 in the TRP family also acts to downregulate macrophage-derived foam cell formation in ApoE−/− mice. This evidence suggest that mechanical force can not only affect the phenotype of macrophages, but also the lipid metabolism of macrophages through mechanical-sensor, and finally further influence the formation of foam cells and the progression of atherosclerosis.

### Lipid metabolism by VSMCs

Studies have shown that mechanical stretching of resting rat VSMCs *in vitro* induces ERK1/2 and leads to accelerated VSMCs proliferation through the LOX-1 signaling pathway (Zhang et al., 2013). Gu et al. (2019) co-cultured VSMCs, ECs, and monocytes using a microfluidic device and applied stretch to them to obtain foam cells. Although the source of the foam cells is still unclear, stretch is known to be very important for the foam cell formation.

Blood flow shear stress also plays an important role in the lipid metabolism of VSMCs. Proprotein convertase subtilisin/kexin type (PCSK9) is a key protein in low-density lipoprotein cholesterol (LDL-C) metabolism and plays a critical role in the degradation of LDL receptors (Lambert et al., 2012). Compared to physiological shear stress, high shear stress inhibited PCSK9 expression in VSMCs, whereas low shear stress or OSS induced PCSK9 expression in VSMCs (Ding et al., 2020). Ding et al. (2015) found that PCSK9 was more highly expressed at the aortic arch bifurcation and the aorta-iliac artery bifurcation, locations with lower shear stress and more susceptible to atherosclerosis; more importantly, low shear stress may induce PCSK9 expression through the NADPH oxidase-dependent ROS production. Currently, the effect of shear stress on PCSK9 remains unclear, which is worthy to be further studied. Furthermore, elevated blood flow shear stress can promote the proliferation and migration of VSMCs through the hydrogen peroxide (H_2_O_2_)-mediated NOX-AKT-SVV axis, which eventually leads to intimal thickening (Zhang et al., 2018), ultimately promoting the development of atherosclerosis.

### ECs

Vascular ECs are in direct contact with blood flow and are regulated by shear stress. The shear stress can influence the CD36 expression in ECs. Shear stress downregulates CD36 expression in a time-and force-dependent manner and is reversible by restoration of no-flow conditions (Bongrazio et al., 2006). LOX-1 was upregulated 9-fold by shear stress (20 dyn/cm^2^, 6 h) in vascular ECs cultured *in vitro* (Nagase et al., 1998). Another study showed that the 5′-regulatory region of LOX-1 contains several potential cis-regulatory elements, such as the GATA-2 binding element, the c-ets-1 binding element, the 12-O-tetradecanoylforbol 13-acetate response element, and the shear stress response element, and that the presence of these cis-acting elements may be responsible for the ability of LOX-1 to respond to shear stress (Aoyama et al., 1999). In addition, SR-B1 expression was increased after high shear stress (15 dyn/cm^2^) stimulation (Yu et al., 2011). Recently, studies have shown that shear stress can regulate the ECs function through the SR-B1-eNOS signaling pathway, thereby influencing the progression of atherosclerosis (Zhang et al., 2016). Thus, regulation of EC SRs by flow shear stress may play a critical role in the formation of EC-derived foam cells.

Therefore, macrophages, VSMCs and ECs are presented in atherosclerotic plaques. Many receptors on these cells that are closely associated with lipid metabolism are also mechanosensors, such as TRPV1, TRPV4 and TRPA1 in macrophages, PCSK9 in VSMCs, and SR-B1, LOX-1 and CD36 in ECs (Figure 2). These elements may affect lipid metabolism in response to altered mechanical forces.

## Summary and perspective

Foam cells plays a vital role in the development of atherosclerosis, which is thought to be differentiated from macrophages, VSMCs, and possibly vascular ECs (Figure 1).

SRs, including SR-A receptor, CD36 and LOX-1, are the major regulators of lipid uptake by macrophages. Upregulation of these receptors can increase lipid uptake by macrophages and thus increase foam cell formation. Upregulation of ACAT1 and downregulation of NCEH can be observed in atherosclerosis, which can further lead to the conversion of macrophages into foam cells. During atherosclerosis, VSMCs switch from a contractile to a synthetic phenotype and express macrophage markers. Downregulation of ABCA1 is found in lipid-rich VSMCs, which increases cholesterol accumulation. Upregulation of LOX-1 with CD36 in ECs also leads to atherosclerosis. It has been shown that vascular ECs can transform into foam cells. At present, there are few studies on the foaming of ECs, but there is no doubt about the importance of ECs in the development of atherosclerosis, and further research is needed in this direction.

Current studies suggest that the main mechanical forces affecting the progression of atherosclerosis are flow shear stress and circumferential stretch. Shear stress may not only affect the formation of atherosclerotic plaques, but also their stability. Mechanical force also had a regulatory effect on cell foaming. In macrophages, low shear stress not only increases MCP-1 expression and macrophage recruitment, but also affects macrophage polarization and lipid metabolism. After stretching, the proliferation of VSMCs was accelerated. More importantly, studies have shown foam cell formation after co-culture of VSMCs, ECs, and monocytes after stretching. Blood flow shear stress also played a regulatory role in the lipid metabolism of VSMCs. In ECs, the blood flow shear stress can modulate the expression of SRs such as CD36, LOX-1, SR-B1, as well as the uptake and efflux of lipids (Figure 2).

Although there are evidence for an important role of mechanical force in the foaming of those cells and the formation of atherosclerotic plaques, studies on the role and underlying mechanism are needed to provide new targets for the control of lipid deposition and the treatment of atherosclerosis.
